# Self-powered RNA nanomachine driven by metastable structure

**DOI:** 10.1093/nar/gkz364

**Published:** 2019-05-11

**Authors:** Shungo Kobori, Yoko Nomura, Yohei Yokobayashi

**Affiliations:** Nucleic Acid Chemistry and Engineering Unit, Okinawa Institute of Science and Technology Graduate University, Onna, Okinawa 904 0495, Japan

## Abstract

Many non-coding and regulatory RNA elements have evolved to exploit transient or metastable structures that emerge during transcription to control complex folding pathways or to encode dynamic functions. However, efforts to engineer synthetic RNA devices have mostly focused on the thermodynamically stable structures. Consequently, significant challenges and opportunities exist in engineering functional RNAs that explicitly take advantage of cotranscriptionally generated transient or metastable structures. In this work, we designed a short RNA sequence that adopts a robust metastable structure when transcribed by an RNA polymerase. Although the metastable structure persists for hours at low temperature, it refolds almost completely into the thermodynamically stable structure upon heat denaturation followed by cooling. The synthetic RNA was also equipped with the Broccoli aptamer so that it can bind its ligand and become fluorescent only in the thermodynamically stable structure. We further demonstrated that the relaxation to the thermodynamically stable and fluorescent structure can be catalyzed by a short trigger RNA in a sequence-specific manner. Finally, the RNA architecture was redesigned to sense and respond to microRNA sequences. In summary, we designed RNA nanomachines that can detect an RNA sequence, amplify signal and produce an optical output, all encoded in a single RNA transcript, self-powered by a metastable structure.

## INTRODUCTION

The structural and functional diversity of RNA has inspired designs of numerous synthetic RNA nanostructures and devices of high complexity (1). These synthetic RNA nanosystems are often composed of multiple strands carefully programmed to self-assemble in a defined stoichiometry to form thermodynamically stable complexes. In nature, most RNA transcripts exist as single strands in living cells and their secondary and tertiary structures often play critical roles in functions such as regulation of gene expression, catalysis and molecular recognition. Therefore, understanding the folding process of RNAs is crucial for elucidating their functions. However, prediction of RNA folding *in vivo* is complicated by the directionality of RNA synthesis. As RNA is transcribed in the 5′ to 3′ direction, local secondary structures may form cotranscriptionally which can affect the global folding pathway and kinetics (2).

In fact, natural RNA elements that actively exploit such cotranscriptionally generated structures have been reported. For example, the *Tetrahymena* group I ribozyme, a large non-coding RNA that catalyzes a multistep self-splicing reaction, utilizes transient intermediate structures to coordinate folding of the complex ribozyme structures and the order of the chemical steps leading to splicing (3). In another example, the RNA bacteriophage MS2 exquisitely controls the dosage and timing of its maturation protein translation by delaying the formation of the thermodynamically stable structure in the 5′ leader sequence that hinders ribosome binding (4). This is achieved by a small local hairpin that transiently forms near the 5′ terminus during RNA replication (5). Similarly, involved in the maintenance of plasmid R1, a metastable structure formed in the 5′ untranslated region (UTR) of the *hok* mRNA both inhibits translation and resists antisense targeting by the Sok-RNA (6,7). As the *hok* mRNA is truncated from the 3′ terminus over time, however, the 5′ UTR structure rearranges to allow translation of the toxic protein (Hok). While translation is still suppressed in plasmid-containing cells due to the Sok-RNA, those cells that lack the plasmid (thus the Sok-RNA with a short half-life) express Hok and are killed.

These and other observations (2,8,9) suggest that some (if not the majority of) natural non-coding and regulatory RNA elements have evolved to optimally fold into functional structures cotranscriptionally, and/or to exploit transient or metastable RNA structures that form during transcription. Such kinetically driven structures enable fine-tuned coordination of complex folding pathways or chemical processes (as in the case of the *Tetrahymena* ribozyme), or sophisticated and dynamic regulation of RNA functions (as in the cases of MS2 and *hok/sok* systems).

**Figure 1.**
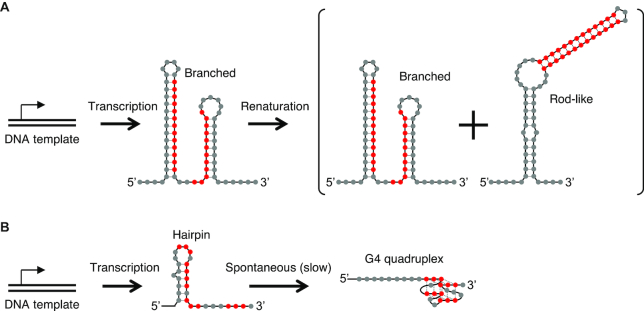
Designed cotranscriptional RNA structures. (**A**) Secondary structures of the ‘direct’ RNA switch by Isambert and coworkers (10). The branched structure was formed almost exclusively by transcription from a DNA template. After renaturation by heating/cooling, the RNA equilibrated to approximately equal amounts of the branched and the rod-like structures. Therefore, the two structures are similar in thermodynamic stability, but the branched structure is kinetically stable when generated cotranscriptionally. The bases that form the central stem in the rod-like structure are shown in red for reference. (**B**) Hairpin/G4 structures by Sugimoto and coworkers (11). Hairpin formation is favored cotranscriptionally, but it slowly and spontaneously relaxes to a G4 structure. The bases involved in G4 are indicated in red.

Synthetic RNA systems that explicitly take advantage of transient and metastable structures that form cotranscriptionally will advance our ability to design sophisticated functional RNAs, as well as our understanding of natural RNA elements. However, very few such systems have been reported. Isambert and coworkers designed simple RNA ‘switches’ that mostly fold into either a rod-like or a branched structure when transcribed *in vitro* (kinetically trapped), but after heat denaturation and cooling, the RNAs equilibrate equally to the two structures with similar thermodynamic stability (Figure 1A) (10).

Another interesting example from the Sugimoto group studied the relaxation of a cotranscriptionally formed hairpin structure to a G-quadruplex (G4) using fluorescent probes (Figure 1B) (11). The hairpin structure was designed to compete with the more thermodynamically stable G4 structure, but the metastable hairpin was formed cotranscriptionally due to sequential folding. However, the transient hairpin structure slowly but spontaneously refolded to G4 in tens of minutes. These groups rationally designed transient or metastable structures that are formed cotranscriptionally and observed their spontaneous or triggered (by heat) relaxation to thermodynamically stable structures.

In this work, we sought to design more sophisticated functions that fully exploit the metastability of cotranscriptionally generated RNA structures. First, we aimed to design a robust metastable structure that can be generated simply by transcription. Second, we wanted the system to fully relax to a thermodynamically stable structure upon appropriate triggers. Finally, the designed RNA should exhibit a prototypical function beyond structural transformation.

## MATERIALS AND METHODS

### Materials

Enzymes, ribonucleotide triphosphates (NTPs) and gel loading buffers were purchased from New England Biolabs (NEB) unless noted otherwise. DFHBI-1T was purchased from Lucerna. All other chemicals used were of molecular biology grade or equivalent, or of the highest quality available, and purchased from Nacalai Tesque or Wako Pure Chemical Co. The 1 × reaction buffer used in this work has the following composition: 40 mM Tris–HCl, pH 7.9, 10 mM MgCl_2_, 1 mM dithiothreitol (DTT), 2 mM spermidine, 100 mM KCl.

### RNA preparation

Linear dsDNA templates that encode the T7 promoter sequence followed by the desired transcripts (Supplementary Table S1) were prepared by polymerase chain reaction (PCR) using a sequence verified plasmid containing the appropriate sequence. The PCR products were purified by agarose gel electrophoresis and silica column purification (Zymoclean Gel DNA Recovery kit, Zymo Research). *In vitro* transcription was performed in a volume of 20 μl containing the 10 × supplied buffer (2 μl), 12.5 mM (each) NTP mix (3.2 μl), 40 mM MgCl_2_ (1 μl), RNase Inhibitor, Murine (0.8 μl), DNA template (400 ng) and T7 RNA polymerase (1 μl). The reactions were kept at 37°C for 3 h. SK-miR-21 was prepared similarly except with 200 ng DNA template, without additional MgCl_2_, and 1 h reaction time. After *in vitro* transcription, DNase I (2.5 μl) was directly added and incubated at 37°C for 15 min to remove DNA template. RNAs were then purified by column (RNA Clean & Concentrator-5 kit, Zymo Research), and concentrations were calculated based on absorbance at 260 nm.

### Denaturing polyacrylamide gel electrophoresis of wMT8

wMT8 RNA samples (53, 106 and 212 ng) were mixed with RNA Loading Dye, (2×) and heated to 95°C for 3 min followed by cooling on ice. The samples were separated on an 8% polyacrylamide gel (acrylamide: bis-acrylamide 19: 1) in Tris-borate-EDTA (TBE) and 8 M urea at 40 V/cm for 25 min. The gel was stained with SYBR Gold (Invitrogen) and imaged by LuminoGraph II (ATTO).

### Native polyacrylamide gel electrophoresis of wMT8

An electrophoresis cell, native 8% acrylamide gel (acrylamide: bis-acrylamide 19: 1), and 1 × TBE buffer were cooled at 4°C for at least 1 h before sample loading. wMT8 RNA was transcribed and column purified as described above. Two 9 μl aliquots of wMT8 RNA (430 ng) diluted in 1.11 × reaction buffer were prepared. One aliquot was heated to 75°C for 5 min and slowly cooled to 37°C using a thermal cycler while the other aliquot was kept on ice. After adding 1 μl H_2_O, the samples were diluted with 1 × reaction buffer to 50, 100 or 200 ng in 4.6 μl. The samples were mixed with 4.6 μl of 2× native gel loading buffer (1:4 mixture of Gel Loading Dye, Purple (6×), no sodium dodecyl sulphate (NEB) and 50% sucrose) and gently loaded onto the precooled gel. The RNAs were separated at 38 V/cm for 25 min at 4°C and was stained with SYBR Gold and imaged by LuminoGraph II.

### End-point fluorescence analysis of the metastable RNAs

Column purified metastable RNAs (0.5 μM) and DFHBI-1T (5 μM) were diluted in the reaction buffer. An aliquot of each solution was renatured by heating to 75°C for 5 min and slowly cooling to 37°C in a thermal cycler. RNA samples (10 μl) were transferred to a 386-well black microplate and fluorescence was measured by an Infinite M1000 PRO microplate reader (Tecan) with excitation at 469 nm, emission at 501 nm and 5 nm bandwidth. The samples were stored at 4°C for 17 h and measured similarly.

### Catalytic relaxation reactions of metastable RNAs

Column purified metastable RNA (1.11 μM) was diluted in 1.11 × reaction buffer with DFHBI-1T (11.1 μM). Nine microliters of the RNA/DFHBI-1T mixture was mixed with 1 μl of a 10 × trigger RNA solution (0–1 μM) in a PCR tube. For the positive controls representing the 1 μM fully thermodynamically stable structures, the metastable RNA (1 μM) was mixed with 1.5 μM each of the stem III binding oligonucleotides (Supplementary Figure S1) and renatured by heating as described above. DFHBI-1T (10 μM) solution in the reaction buffer was used as the negative control to subtract any background fluorescence. Fluorescence of Broccoli-DFHBI-1T complex was monitored at 37°C using a real-time PCR instrument (StepOnePlus, Applied Biosystems).

### Relaxation of wMT8 by c4+8 during transcription

wMT8 DNA template solution (100 ng, 1.3 μl), 2.5 × reaction buffer (4 μl), 25 mM (each) NTP mix (0.8 μl), 50 μM DFHBI-1T (2 μl), RNase Inhibitor, Murine (0.4 μl), and T7 RNA polymerase (0.5 μl) were mixed on ice (total 9 μl). To this solution was added 1 μl of 500 nM c4+8 or c4+8sc solution. Water (1 μl) was added for the uncatalyzed reaction. Broccoli-DFHBI-1T fluorescence of these samples were monitored at 37°C using a real-time PCR instrument (StepOnePlus, Applied Biosystems).

## RESULTS

### Design of metastable RNAs

**Figure 2.**
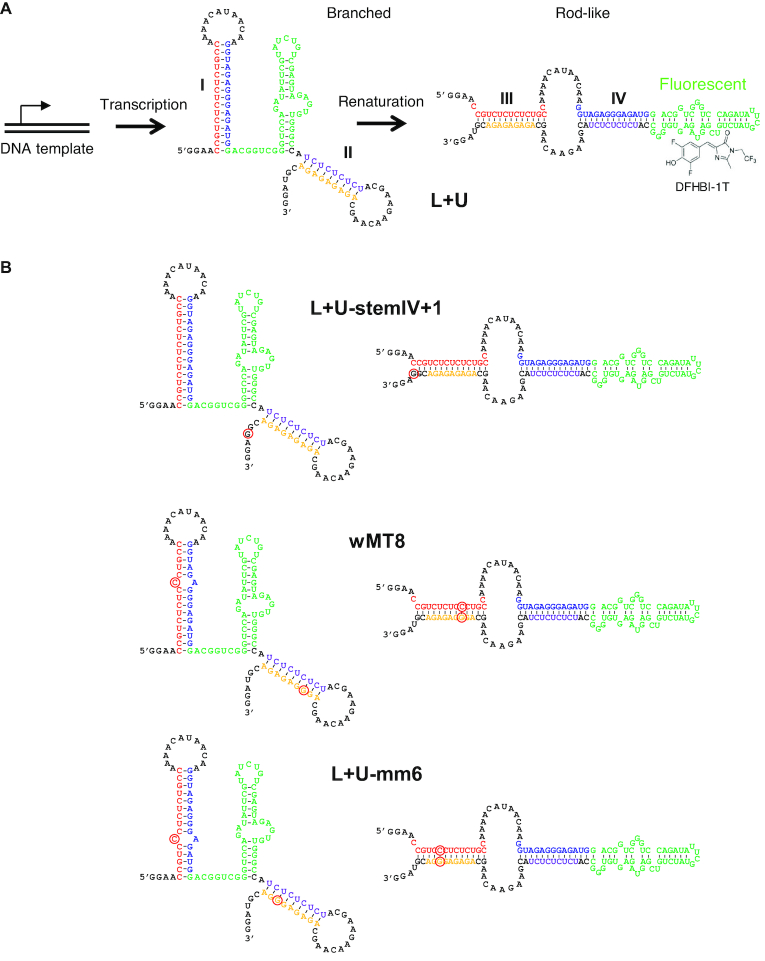
Proposed secondary structures of the metastable RNAs. (**A**) The majority of the L+U transcript adopts the branched structure because stem I is formed first cotranscriptionally. The branched structure is metastable (kinetically stable) and is robust enough to maintain its structure for 17 h at 4°C. Renaturation by heating and cooling transforms most of the RNA to the rod-like structure with stems III and IV. The Broccoli aptamer (green) can bind DFHBI-1T and fluoresce only in the rod-like structure. (**B**) Variants of L+U intended to increase the relative stability of the rod-like structure compared to the branched structure. Introduced mutations are indicated by red circles. L+U-stemIV+1 harbors a single mutation near the 3′ end that extends stem IV by 1 bp. wMT8 contains a C-A mismatch within stem I to destabilize the branched structure, with a compensatory mutation near the 3′ end to stabilize stem IV, therefore, the rod-like structure. A similar set of mutations were introduced in L+U-mm6 which contains a mismatch at a different location within stem I.

We based our initial design (Figure 2) on the branched vs rod-like structures of Isambert and coworkers (Figure 1A) (10). Our goal was to make the rod-like structure more thermodynamically stable, but to make the branched structure kinetically stable (metastable) during transcription. Moreover, we incorporated the Broccoli aptamer (12) in the central loop region so that the aptamer would be functional only in the rod-like (thermodynamically stable) structure. The Broccoli aptamer binds the RNA-dependent fluorophore DFHBI-1T and becomes fluorescent, enabling us to monitor the structural transition in real time. As the RNA is transcribed, stem I should form first, followed by stem II to yield the branched structure. The key design constraint is to prevent an intramolecular strand exchange reaction that leads to the formation of stems III and IV resulting in the thermodynamically stable rod-like structure. Strand exchange reactions are greatly accelerated by so-called single-stranded toehold regions that can initiate strand exchange. While toehold mediated strand exchange reactions have been extensively studied and exploited in multistrand DNA and RNA systems (13), controlling such reactions within a polynucleotide requires more stringent design and experimentation.

We started with a design shown in Figure 2A (L+U). Based on this structure, several additional variants were designed with an intention to further stabilize the rod-like structure relative to the branched structure (Figure 2B). In L+U-stemIV+1, a single base mutation near the 3′ end of L+U was expected to increase the size of stem IV by 1 bp without affecting stems I and II in the branched structure. In wMT8, a C-A mismatch was introduced to destabilize stem I in the branched structure as well as a compensatory mutation near the 3′ end that replaces a U-A pair in stem IV with a C-G pair, thereby stabilizing the rod-like structure. A similar set of mutations were introduced in L+U-mm6 resulting in a mismatch in a different location within stem I (Figure 2B). Through these designs, we sought to represent structures with different relative stabilities of the rod-like vs branched structures, as well as different local stabilities of the stem structures. Additionally, we tried to avoid apparent single-stranded toehold regions in the branched structure that can trigger a spontaneous strand exchange reaction to yield the rod-like structure.

### Thermal relaxation of metastable RNAs

**Figure 3.**
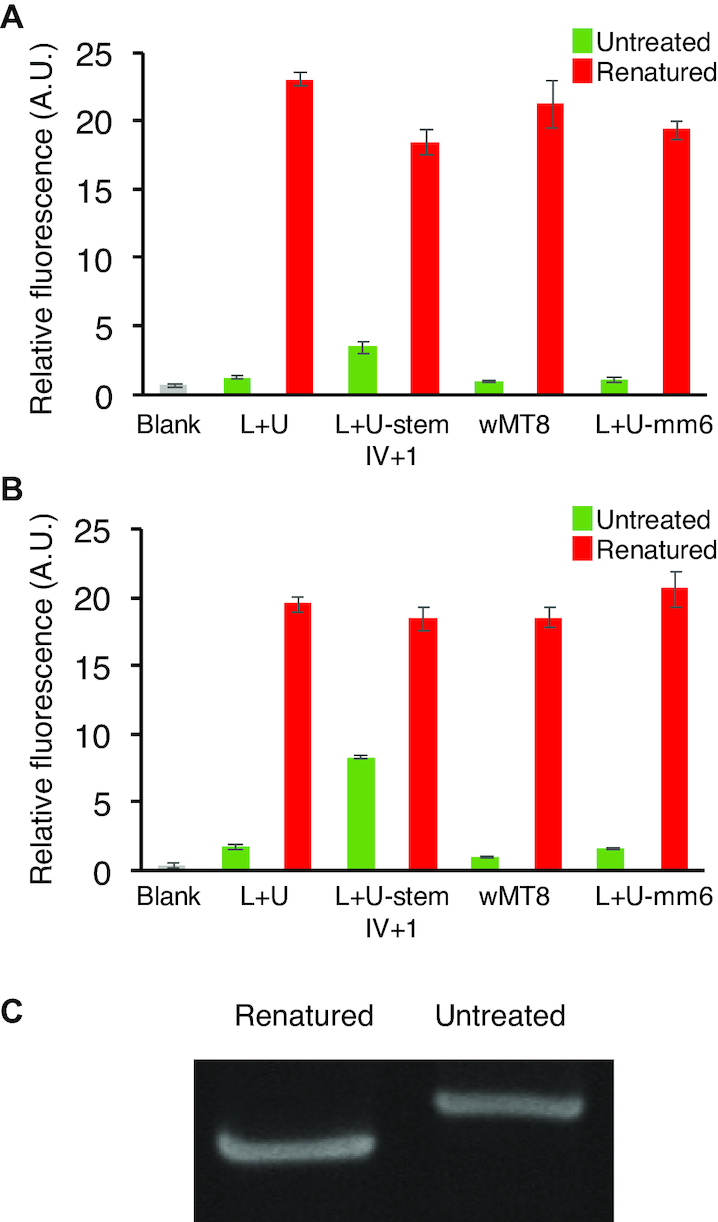
Characterization of metastable and thermodynamically stable RNA structures. (**A**) Fluorescence of the metastable RNAs (500 nM) generated by *in vitro* transcription with and without renaturation after heating. The solutions contain 5 μM DFHBI-1T. The blank contains the buffer and DFHBI-1T. Fluorescence values were normalized to that of the untreated wMT8 with DFHBI-1T (1.0). The data are averages of three replicate samples with the error bars representing S.D. (**B**) Fluorescence of the same samples in (A) after 17 h kept at 4°C. (**C**) Native gel electrophoresis of *in vitro* transcribed wMT8. An aliquot was kept on ice (left lane), and another aliquot was renatured by heating to 75°C followed by slow cooling.

We synthesized the designed metastable RNAs (Figure 2) by *in vitro* transcription from appropriate DNA templates using T7 RNA polymerase. The reactions were stopped after 3 h and treated with DNase I. The RNAs were then purified by silica columns and diluted to 500 nM in the reaction buffer supplemented with 5 μM DFHBI-1T. An aliquot from each sample was renatured by heat to convert the metastable RNA to the thermodynamically stable structure. The Broccoli fluorescence measurements of the renatured and untreated RNAs showed higher fluorescence in the renatured samples in all RNA designs (Figure 3A). However, the untreated L+U-stemIV+1 showed higher fluorescence compared to the other designs, indicating a lower kinetic stability of the branched structure. The branched structures of the untreated L+U, wMT8, and L+U-mm6 were remarkably robust, showing little signs of relaxation after 17 h incubation at 4°C while fluorescence of the untreated L+U-stemIV+1 sample increased significantly (Figure 3B).

We further analyzed wMT8 by gel electrophoresis. Analysis by denaturing polyacrylamide gel electrophoresis (PAGE) showed a major band of the expected size (129 nt) with few minor bands attributable to prematurely truncated products (Supplementary Figure S2A). The untreated and renatured wMT8 were analyzed by native PAGE. The untreated and renatured RNAs showed clearly distinct mobility with the untreated (presumably branched) RNA moving slower than the (presumably rod-like) renatured RNA (Figure 3C and Supplementary Figure S2B).

### Catalytic relaxation of metastable RNAs by RNA

**Figure 4.**
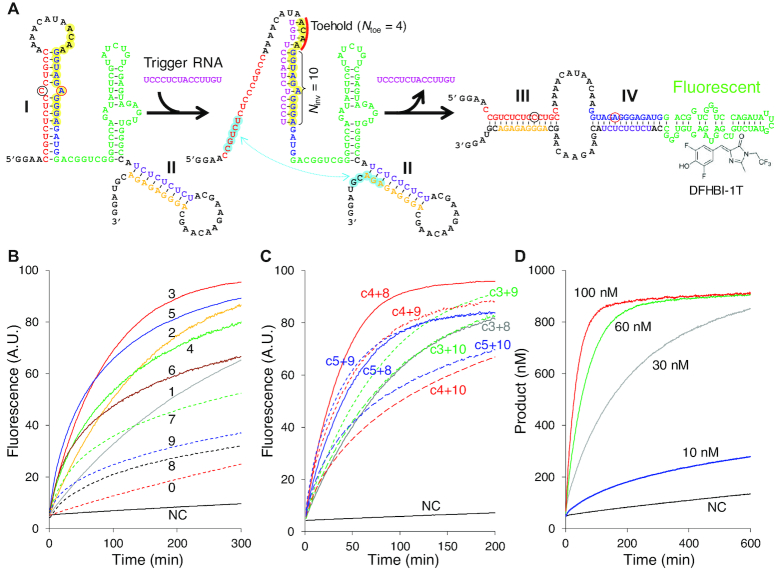
Catalytic relaxation of wMT8 by a trigger RNA. (**A**) Mechanism of catalytic transformation of wMT8 from the metastable to the thermodynamically stable structure by a trigger RNA (c4+10 shown). The sequence complementary to the trigger RNA is highlighted in yellow, with the toehold region marked with a red line. The internal toehold region that is transiently activated by the trigger RNA hybridization is highlighted in blue. (**B**–**D**) RNA triggered catalytic wMT8 relaxation monitored via Broccoli-DFHBI-1T fluorescence. All reactions contained 1.0 μM wMT8, 10 μM DFHBI-1T. Trigger RNA concentration was 100 nM unless specified otherwise. (B) Variation of toehold length in c*N*_toe_+10 where *N*_toe_ = 0–9. The numbers indicate *N*_toe_. NC: no catalyst. (C) Varying the invading strand length (*N*_inv_ = 8, 9, 10) for *N*_toe_ = 3, 4, or 5. (D) wMT8 response to different concentrations of c4+8. Data shown are averages of two replicates.

Having established that L+U, wMT8 and L+U-mm6 adopt robust metastable structures after transcription and that the thermodynamically stable structure activates the Broccoli aptamer, we set out to explore means other than heating/renaturation to trigger the structural transformation. An intriguing possibility was to use an RNA trigger to transiently destabilize a stem in the branched structure to create a toehold from which an intramolecular strand exchange reaction can be initiated. For example, the short trigger RNA (c4+10) shown in Figure 4A can invade stem I to expose the 5′ end of stem I (highlighted in blue) which can serve as a toehold to form stem III of the rod-like structure. Furthermore, the trigger RNA would be expelled upon formation of the rod-like structure, allowing it to catalyze additional structural transformations. Therefore, the trigger RNA serves as a sequence-specific catalyst for the refolding process. Alternatively, the metastable RNA can be viewed as a unimolecular RNA nanomachine that can detect RNA, amplify signal and produce an optical output, fueled solely by the refolding energy of the metastable structure to the thermodynamically stable structure.

First, the three robust metastable RNAs (1 μM) were mixed with DFHBI-1T (10 μM) and the trigger RNA c4+10 (100 nM) in the reaction buffer, and the Broccoli fluorescence was monitored over time at 37°C (Supplementary Figure S3). Interestingly, the metastable RNAs showed distinct responses to the RNA trigger. wMT8 showed a sharp increase in fluorescence, indicating its efficient conversion to the thermodynamically stable structure by c4+10. L+U-mm6 which differs from wMT8 by the position of the mismatch within stem I was converted less efficiently. L+U without a mismatch in stem I showed only modest conversion by the c4+10. Consequently, we decided to focus on wMT8 for further analysis.

First, we systematically varied the trigger RNAs. The trigger RNAs were named c*N*_toe_+*N*_inv_ where *N*_toe_ is the number of bases that hybridize with the loop sequence (toehold), and *N*_inv_ is the number of bases that hybridize with (invade) the 3′ side of stem I. Fixing *N*_inv_ to 10 bases, the size of the toehold (*N*_toe_) was varied from 0 to 9. Metastable wMT8 (1 μM) was mixed with DFHBI-1T (10 μM) and a trigger RNA (100 nM), and the Broccoli fluorescence was monitored over time at 37°C (Figure 4B). The results indicate that the initial rate increases as *N*_toe_ increases up to 5 (Supplementary Figure S4A) which suggests that toehold binding is the rate limiting step under the condition.

Interestingly, slow catalysis was observed even without the toehold (*N*_toe_ = 0) probably due to spontaneous ‘breathing’ of the stem near the loop region. While relaxation of the metastable wMT8 to the thermodynamically stable structure was mostly complete within 300 min (for *N*_toe_ = 2–5), slower catalysis and lower turnover was observed for *N*_toe_ = 8 and 9 likely due to slower release of the trigger RNA. Overall, toehold size between 3 and 5 showed faster initial rate and higher conversion among the tested trigger RNAs.

We next compared trigger RNAs with *N*_toe_ = 3–5 and *N*_inv_ = 8–10. Among them, c4+8 stood out in both the fast initial rate and high conversion after ∼200 min (Figure 4C and Supplementary Figure S4B). Therefore, wMT8 relaxation in the presence of varying concentrations of c4+8 was monitored quantitatively (Figure 4D) using fully renatured wMT8 as a reference (Supplementary Figure S1). Approximately 5% of the transcript (50 nM) existed as the thermodynamically stable state at the start of the reaction. In the presence of c4+8 at 60 nM or higher, up to 900 nM of the fluorescent structure was obtained after 300 min. The remaining 100 nM wMT8 may assume nonproductive secondary structures. The maximum catalyst turnover was observed toward the end of the reaction when each catalyst molecule converted ∼24 molecules of wMT8 from the metastable to the thermodynamically stable state in the presence of 30 nM c4+8. Overall, we demonstrated that short RNAs that are partially complementary to wMT8 can catalyze the relaxation of the kinetically trapped metastable structure to the thermodynamically stable structure. Moreover, complementarity of the trigger RNA influences the kinetics of the relaxation reaction.

### Relaxation of wMT8 by c4+8 during transcription

We examined if wMT8 can be relaxed during transcription in the presence of the c4+8 trigger. wMT8 was transcribed *in vitro* in the presence of DFHBI-1T (10 μM) and c4+8 (50 nM). The results (Supplementary Figure S5) indicate efficient conversion into the fluorescent, thermodynamically stable structure *in situ*. The catalytic activation is sequence specific, as indicated by the reaction in the presence of a scrambled trigger RNA sequence (c4+8sc, Supplementary Table S1) was indistinguishable from the uncatalyzed reaction (Supplementary Figure S5).

### Reconfiguring the metastable RNA architecture to sense microRNA sequences

**Figure 5.**
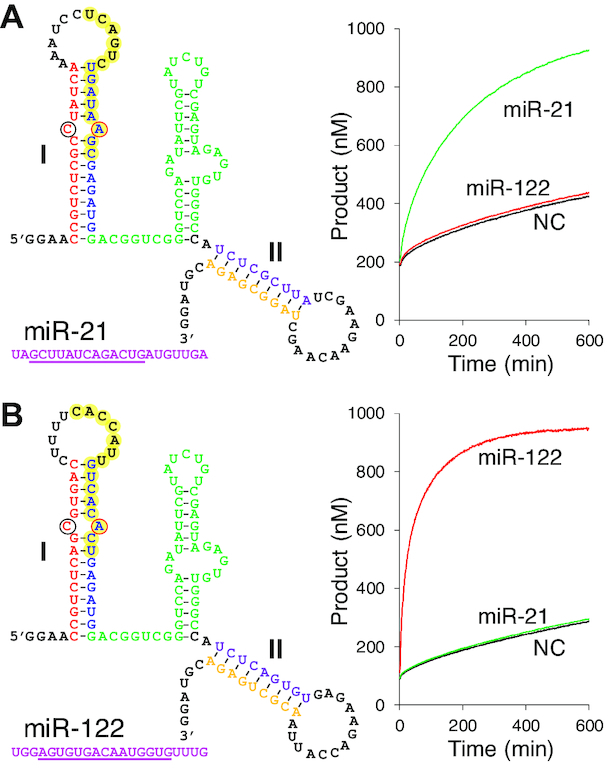
Metastable RNA sensors designed to detect microRNA sequences. The part of miRNA sequence (pink) that hybridize with the sensor is underlined. The sensor sequence complementary to the targeted microRNA is highlighted in yellow. The graphs indicate the fluorescence responses of the sensors (1 μM). Trigger RNA concentration was 100 nM. NC: no catalyst. (**A**) miR-21. (**B**) miR-122. Data shown are averages of three replicates.

We next examined the possibility of adapting the wMT8 architecture to respond to alternative trigger RNA sequences. This required redesigning the ‘sensor’ sequence, or the part of wMT8 that is complementary to the trigger RNA. Alteration of the sensor sequence further necessitates modifications in other parts of the RNA to maintain the overall structure and relative thermodynamic stabilities of the metastable and the thermodynamically stable states. A general diagram showing the variable and the core positions of the metastable RNA architecture is shown in Supplementary Figure S6. This turned out to be a nontrivial problem requiring iterations of design, synthesis and testing. While a more systematic and predictable design process is our future goal, we successfully adapted the wMT8 architecture to sense and respond to two microRNA sequences miR-21 (SK-miR-21) and miR-122 (SK-miR-122) (Figure 5). SK-miR-21 was catalytically converted to the fluorescent thermodynamically stable state by miR-21 but not by miR-122, demonstrating the specificity of the platform (Figure 5A). Similarly, SK-miR-122 was only responsive to miR-122 (Figure 5B).

## DISCUSSION

Dependence of proper RNA folding on cotranscriptional conditions has been observed in many natural RNA elements such as in the *Tetrahymena* group I intron (3). Therefore, the primary sequences of many functional RNAs encode not only the functional structures, but also other dynamic properties such as folding pathways and transient or metastable structures (2,8,9,14). Inspired by nature, synthetic functional RNAs such as riboswitches have been designed with sophisticated functions (15–17). However, such efforts have mostly focused on the thermodynamically stable structures and have paid little or no attention to exploiting transient or metastable structures that emerge during transcription. In this work, we designed synthetic RNA sequences that adopt a robust metastable secondary structure via cotranscriptional folding. This metastable branched structure can subsequently be refolded almost entirely into a thermodynamically stable rod-like structure, either via heat induced renaturation or catalytically by a short trigger RNA.

Synthetic multistrand DNA and RNA systems that perform complex molecular computation including signal amplification have been extensively investigated (18–25). The Spinach and Broccoli aptamers have also been extensively used as RNA-based sensor and circuit outputs (22,26–30). These systems are driven by the hybridization energy and/or the increase in entropy upon complexation of multiple oligonucleotides to perform various functions. The process often involves an excess ‘fuel strand’ to drive the strand displacement reactions which ultimately forms an unproductive waste complex with another strand. Spontaneous relaxation to the final thermodynamically stable complexes must be precluded by careful design and preparation of the individual sequences to control the availability of toeholds that can initiate the strand exchange reactions. Many of these systems require separate production and folding of individual components which are then mixed in a defined stoichiometry for optimal function. Our system, on the other hand, is a unimolecular system driven solely by the refolding energy of a robust metastable structure which is automatically formed during transcription. Moreover, the wMT8 system described here is ‘wasteless’ in the sense that the relaxed state is not merely a thermodynamic sink, but also serves as a fluorescence reporter. Simplicity of the unimolecular architecture and its direct formation via transcription may also be advantageous for future applications *in vivo*.

Based on the wMT8 design, we successfully adapted the metastable RNA to respond to two distinct microRNA sequences. However, the catalytic efficiency was not as high as that of wMT8/c4+8 and faster uncatalyzed relaxation reactions were observed (Figure 5). As an alteration of the sensor sequence constrains other parts of the metastable RNA structure (Supplementary Figure S6), it can lead to undesirable consequences that disrupt the relative stability of the two structures. There are several strategies to minimize the uncatalyzed relaxation, for example, by increasing the stability of stems I and II in the metastable structure, especially near the putative internal toehold regions. Additional studies to identify the key structural requirements of the metastable RNA will be needed to more efficiently engineer the system to sense new RNA sequences. It may also be desirable to develop a high-throughput screening method to gain a more comprehensive and quantitative understanding of the sequence–function relationship.

It is known that some complex RNA structures such as the group I intron are prone to misfold into kinetically trapped structures. It may be worthwhile to investigate if small RNA or oligonucleotide triggers can be designed to correct misfolded or metastable RNA structures found in nature or designed artificially.

Our ability to encode multiple functions (sensing, signal amplification and optical output) with a built-in energy source into single RNA molecules opens a new path for design of biomolecular machines. Future goals include designing self-powered RNA nanomachines with more than one metastable state, with multiple sensors, or with other computational or output functions.

## Supplementary Material

gkz364_Supplemental_File
