# Supplementary material for: Self-powered RNA nanomachine driven by metastable structure
Source: Nucleic Acids Res. 2019 May 11;47(11):6007–14. doi: 10.1093/nar/gkz364 (PMC6582335; doi:10.1093/nar/gkz364)
Supplement: gkz364_Supplemental_File [file gkz364_supplemental_file.pdf]

Supplementary Data

Self-powered RNA nanomachine driven by metastable structure

Shungo Kobori, Yoko Nomura and Yohei Yokobayashi*

Nucleic Acid Chemistry and Engineering Unit, Okinawa Institute of Science and Technology Graduate University, Onna, Okinawa, 904 0495, Japan

*For correspondence: yohei.yokobayashi@oist.jp

Table S1. RNA Sequences[a]

L+U[b]	GGAACCGUCUCUCUCUGCCAAAACAUAACAAGGUAGAGGGAGAUGGACGGUCGGGUCCAGAUAUUC<br>GUAUCUGUCGAGUAGAGUGUGGGCCAUCUCUCUCUACGAAGAACAAGCAGAGAGAGACGUAGG
L+U-stemIV+1[b]	GGAACCGUCUCUCUCUGCCAAAACAUAACAAGGUAGAGGGAGAUGGACGGUCGGGUCCAGAUAUUC<br>GUAUCUGUCGAGUAGAGUGUGGGCCAUCUCUCUCUACGAAGAACAAGCAGAGAGAGACG**G**AGG
wMT8[b]	GGAACCGUCUCUC**C**CUGCCAAAACAUAACAAGGUAGAGGGAGAUGGACGGUCGGGUCCAGAUAUUC<br>GUAUCUGUCGAGUAGAGUGUGGGCCAUCUCUCUCUACGAAGAACAAGCAG**G**GAGAGACGUAGG
L+U-mm6[b]	GGAACCGUC**C**CUCUCUGCCAAAACAUAACAAGGUAGAGGGAGAUGGACGGUCGGGUCCAGAUAUUC<br>GUAUCUGUCGAGUAGAGUGUGGGCCAUCUCUCUCUACGAAGAACAAGCAGAGAG**G**GACGUAGG
SK-miR-21[b]	GGAACCGUCUCGCCUAUCAAAAUCCUCAGUCUGAUAAGCGAGAUGGACGGUCGGGUCCAGAUAUUC<br>GUAUCUGUCGAGUAGAGUGUGGGCCAUCUCGCUUAUCGAAGAACAAGCUAGGCGAGACGUAGG
SK-miR-122[b]	GGAACCGUCUCAGCGUGACCUUUUCACCAUUGUCACACUGAGAUGGACGGUCGGGUCCAGAUAUUC<br>GUAUCUGUCGAGUAGAGUGUGGGCCAUCUCAGUGUGAGAAGACCAUUAACGCUGAGACGUAGG
c0+10	UCCCUCUACC
c1+10	UCCCUCUACCU
c2+10	UCCCUCUACCUU
c3+10	UCCCUCUACCUUG
c4+10	UCCCUCUACCUUGU
c5+10	UCCCUCUACCUUGUU
c6+10	UCCCUCUACCUUGUUA
c7+10	UCCCUCUACCUUGUUAU
c8+10	UCCCUCUACCUUGUUAUG
c9+10	UCCCUCUACCUUGUUAUGU
c3+8	CCUCUACCUUG
c3+9	CCCUCUACCUUG
c4+8	CCUCUACCUUGU
c4+9	CCCUCUACCUUGU
c5+8	CCUCUACCUUGUU
c5+9	CCCUCUACCUUGUU
miR-21	UAGCUUAUCAGACUGAUGUUGA
miR-122	UGGAGUGUGACAAUGGUGUUUG
c4+8sc	UGUUCCAUCUCC

[a]All sequences are shown in 5’ to 3’ direction. [b]CCATAATACGACTCACTATA (T7 promoter underlined) was added upstream of the corresponding DNA sequences in the dsDNA templates for *in vitro* transcription.

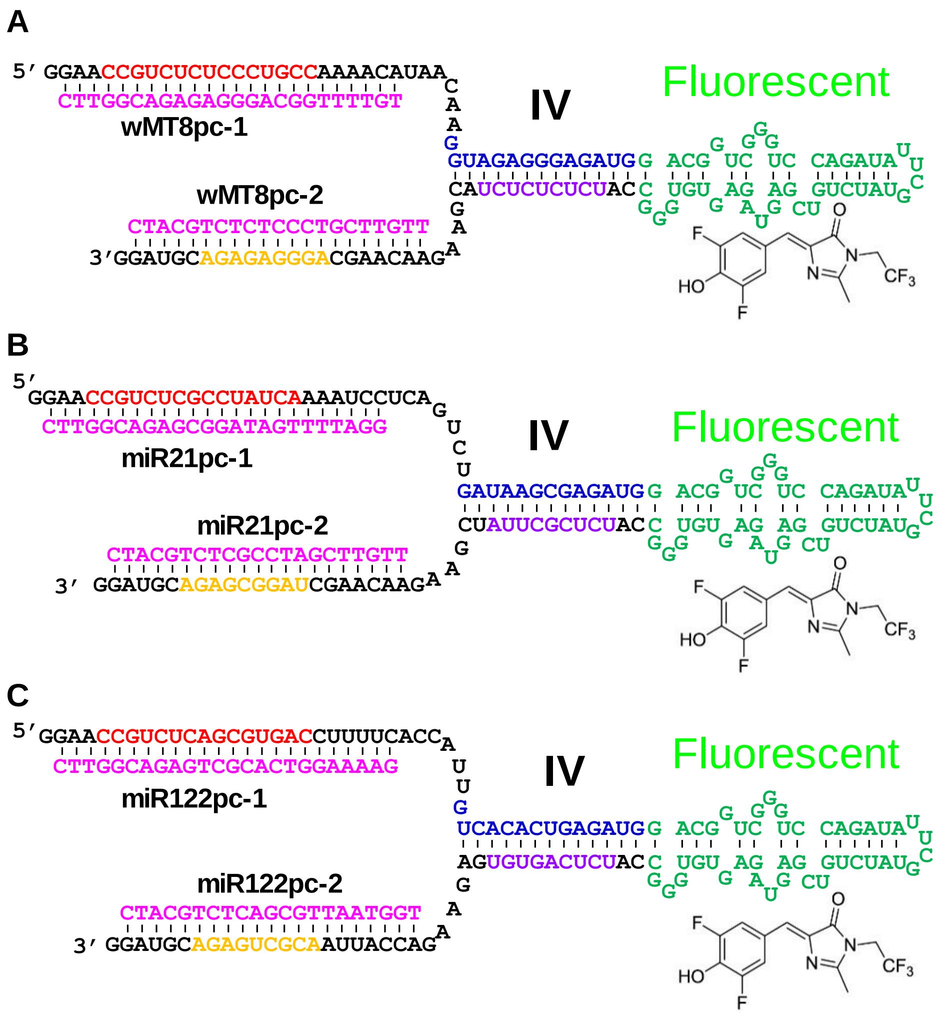

Figure S1. Positive controls used to normalize the concentrations of the fluorescent thermodynamically stable structures in the catalytic relaxation reactions (Figure 4D and Figure 5). Each RNA (1 μM) was mixed with 1.5 μM of two oligo DNAs shown (pink) and denatured by heating to 75 ºC and slowly cooled to RT. The oligonucleotides were designed to inhibit stem I and stem II formation, while allowing stem IV to form. (**A**) wMT8. (**B**) SK-miR-21. (**C**) SK-miR-122.

A

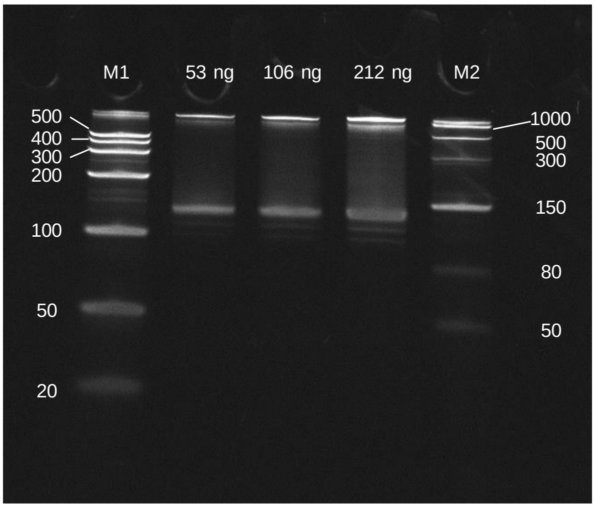

B

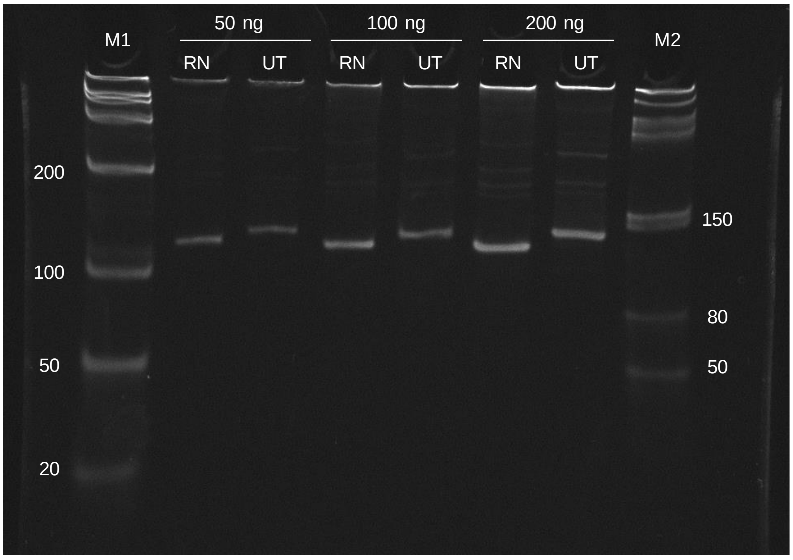

Figure S2. Analysis of wMT8 by 8% PAGE. *In vitro* transcribed wMT8 (129 nt) was treated with DNase I and column purified. (**A**) Denaturing PAGE. RNA samples were heated to 95 °C for 3 min and cooled on ice before loading. Total RNA amounts loaded are indicated above each lane. (**B**) Native PAGE. wMT8 of the amount indicated above each lane was either untreated (UT) or heat renatured (RN) (heated to 75 °C and slowly cooled) prior to loading. M1: DynaMarker RNA Low II (BioDynamics Laboratory). M2: Low Range ssRNA Ladder (NEB). High MW bands (>1000 nt) appear in all wells including those of the size markers, and appear to be due to nonspecific RNA aggregation.

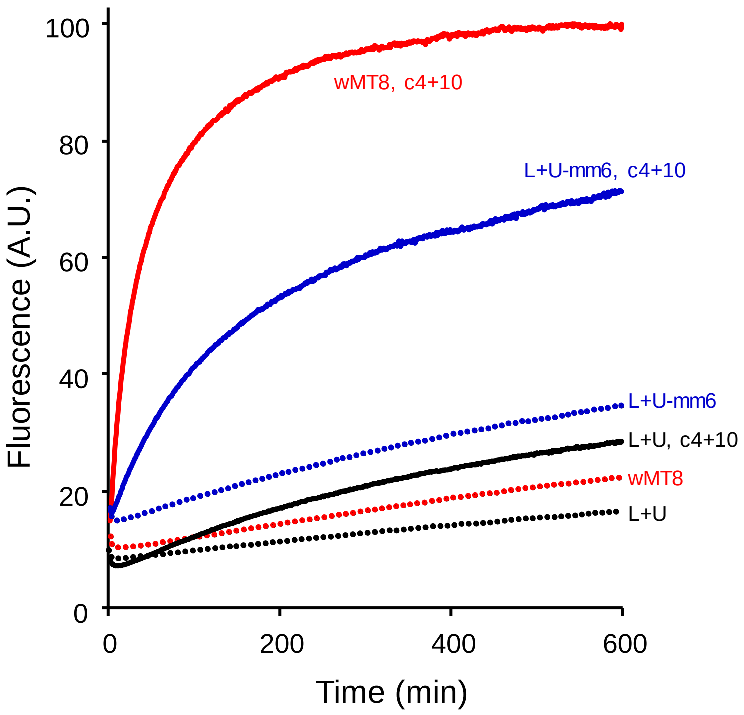

Figure S3. Catalytic relaxation of the metastable RNAs (1 μM L+U, wMT8, or L+U-mm6) in the presence of the trigger RNA c4+10 (100 nM). DFHBI-1T was present at 10 μM. The dotted lines are uncatalyzed reactions and the solid lines represent the catalyzed reactions. The data are averages of duplicate samples.

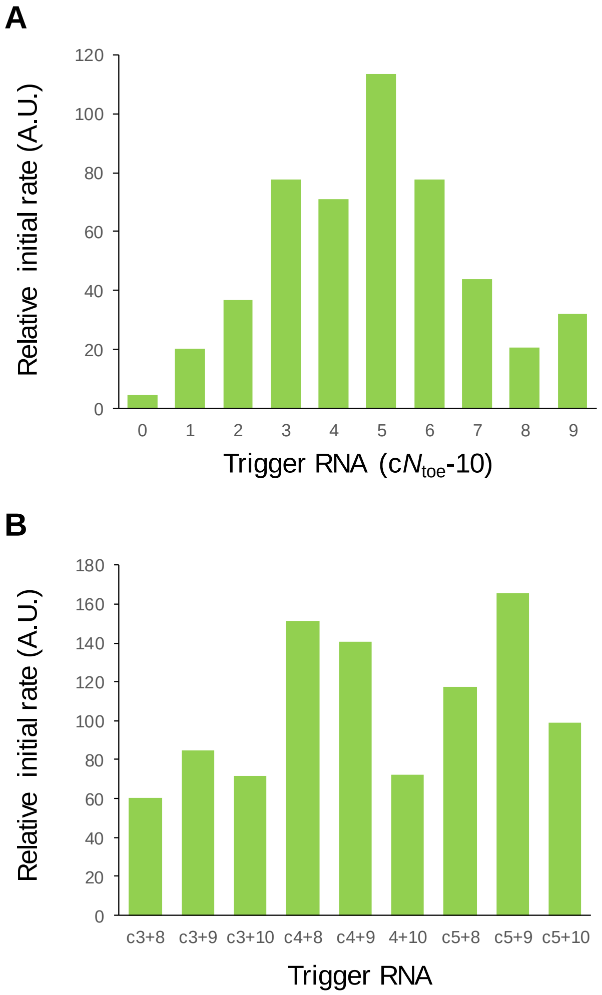

Figure S4. Initial rates of the catalytic relaxation reaction of wMT8 by different trigger RNAs. The rates shown are relative to the uncatalyzed rate. (**A**) Different toehold lengths in cN_{toe}-10 shown in Figure 4B. (**B**) Different invading strand lengths for N_{toe} = 3, 4, or 5 shown in Figure 4C.

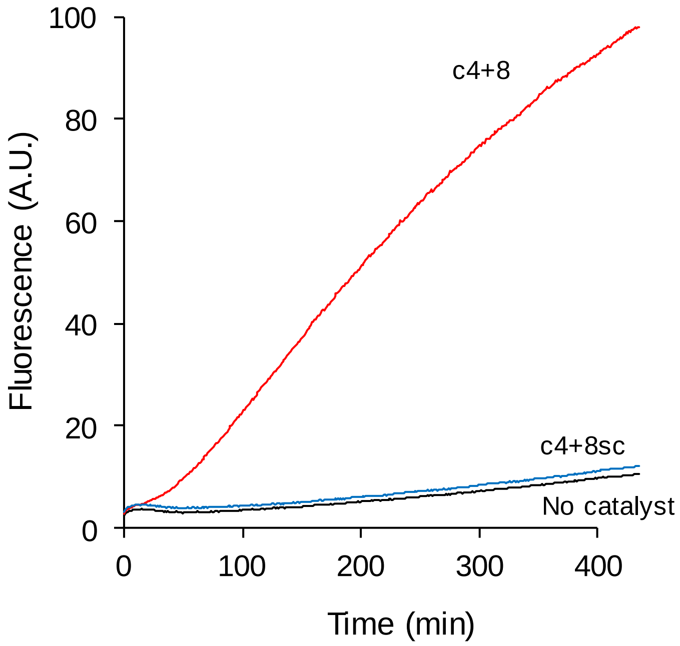

Figure S5. Cotranscriptional activation of wMT8 by c4+8 (50 nM). The background fluorescence of the DFHBI-1T solution without RNA was subtracted from the raw fluorescence values. c4+8sc (50 nM) is a scrambled sequence based on c4+8 used as a nonspecific trigger control (**Table S1**). The data are averages of duplicate reactions.

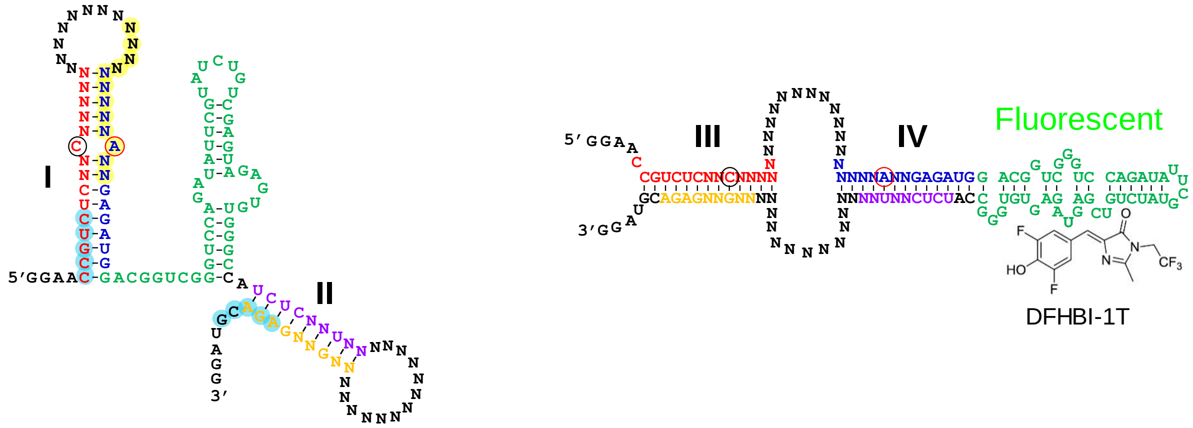

Figure S6. The general architecture of the RNA-responsive metastable RNA. The “N” nucleotides highlighted in yellow (plus or minus several bases in the loop) are varied according to the desired trigger RNA. These bases then constrain the complementary positions in stem I (red Ns) and stem IV (purple Ns and some black Ns in the loop). The red Ns also constrain the orange Ns in stem III as well as few black Ns in the loop. The number of nucleotides and sequences of the loops (black Ns) are also variable. The C-A mismatch in stem I may also be A-C if the corresponding complementary positions in stems III and IV are also modified. This mismatch in stem I (and lack of it in stems III and IV) destabilized the metastable structure relative to the thermodynamically stable structure.
